# From multiple pathogenicity islands to a unique organized pathogenicity archipelago

**DOI:** 10.1038/srep27978

**Published:** 2016-06-15

**Authors:** Costas Bouyioukos, Sylvie Reverchon, François Képès

**Affiliations:** 1 Institute of Systems and Synthetic Biology, Genopole, CNRS, Univ. Evry, 91000 ÉVRY, France; 2 Univ Lyon, Université Lyon 1, INSA-Lyon, CNRS UMR5240, MAP, F-69622 VILLEURBANNE, France; 3 Department of BioEngineering, Imperial College London, United Kingdom

## Abstract

Pathogenicity islands are sets of successive genes in a genome that determine the virulence of a bacterium. In a growing number of studies, bacterial virulence appears to be determined by multiple islands scattered along the genome. This is the case in a family of seven plant pathogens and a human pathogen that, under KdgR regulation, massively secrete enzymes such as pectinases that degrade plant cell wall. Here we show that their multiple pathogenicity islands form together a coherently organized, single “archipelago” at the genome scale. Furthermore, in half of the species, most genes encoding secreted pectinases are expressed from the same DNA strand (transcriptional co-orientation). This genome architecture favors DNA conformations that are conducive to genes spatial co-localization, sometimes complemented by co-orientation. As proteins tend to be synthetized close to their encoding genes in bacteria, we propose that this architecture would favor the efficient funneling of pectinases at convergent points within the cell. The underlying functional hypothesis is that this convergent funneling of the full blend of pectinases constitutes a crucial strategy for successful degradation of the plant cell wall. Altogether, our work provides a new approach to describe and predict, at the genome scale, the full virulence complement.

The analysis of contiguous genome segments across species has highlighted the conservation of gene order (synteny) along short chromosomal stretches^1^,^2^,^3^. More recently, long-range patterns encompassing the genome scale have additionally been uncovered. Studies involving co-regulated^4^,^5^,^6^, co-functional^7^, and evolutionary correlated^8^ gene sets have all identified periodic and proximal patterns formed by these genes along chromosomes of eubacteria, archaebacteria and yeast. Most of these studies on long-range patterns made use of a recently developed algorithm called “*GREAT:SCAN:patterns*”^6^,^9^ that detects genomic patterns with exquisite sensitivity. Here, we applied “*patterns*” to assess whether pathogenicity islands form a coherent array at the genome scale.

Indeed, one of the defining features of pathogenicity islands is the proximity of their constituent genes along the genome sequence. It is generally admitted that this one-dimensional (1-D) proximity is an evolutionarily important characteristic, as it favors a full transplant of the chromosomal pathogenic determinants during a horizontal transfer event. However, it was observed on certain genomes that the expression of pathogenicity relied on several islands that were scattered along the genome^10^. Such a pathogenicity “archipelago” (a term probably coined in 2002^11^,^12^) thus raises unsolved issues of efficient co-transfer, co-action and co-regulation. Here a wide but homogeneous family of archipelagoes is studied through seven plant pathogens belonging to two bacterial genera^13^,^14^ and one human pathogen that can occupy many different environmental habitats including plants^15^ (Table 1). These organisms all share a type II secretory apparatus^16^. It allows the secretion of massive amounts of a blend of bacterial pectinases that together degrade pectin in plant cell walls and result in soft rot^15^,^17^. The genes encoding pectinases, the type II secretory apparatus, membrane transporters, and the intracellular enzymes of pectin catabolism, are co-regulated by the KdgR transcription factor which is responsible for their induction in the presence of pectin^18^. This set of KdgR-regulated genes (KdgR regulon) is widely scattered in many pathogenicity islands along the genomes of these organisms (*e.g., Dickeya dadantii* from Table 1: 19 genes located in 15 distinct loci/islands)^18^,^19^,^20^. Their comparative study thus offers an opportunity to uncover rules for the long-range positioning of such islands.

## Results

**Figure 1:**
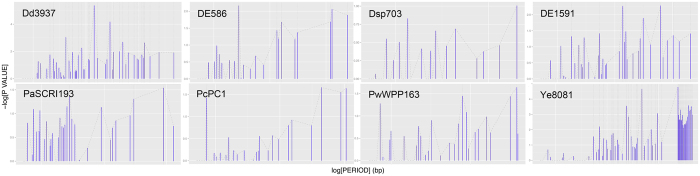
Periodograms of the KdgR regulon for all eight genomes. For each of the eight species (one per panel labelled by its short name), its KdgR regulon dataset was sujected to pattern analysis by a periodicity/proximity detection algorithm^9^ which scans every possible period from 5 kbp to full genome length in the set of positions of the KdgR target genes. Periods are plotted along the X-axis on a logarithmic scale. The significance score, corrected for period-dependance (see Methods), of each period is plotted on the Y-axis. A high peak at period *P* denotes its exceptional corrected significance. Where the peak(s) is on the right end of the spectrum (*e.g.*, Ye8081), *i.e.* for periods close or equal to full genome length or a large fraction of it (one half, one third etc.), it denotes a proximal arrangement of the genes around the circular chromosome. The white vertical gridline marks the 10-kbp, 100-kbp, and if applicable 1-Mbp period values. The dotted vertical lines indicate significant periods, and the dashed line connects the tips of the bars to provide a view of regions with dense periodic signal detection.

To investigate each genomic pattern, the periodicity detection algorithm “*patterns*” scans every possible period in the set of positions of the KdgR target genes. The significance of each period is evaluated by the probability that a similar or superior periodicity level could be achieved with a randomized set of positions. This probability is then corrected differentially to become period-independent. Indeed, short periods give numerous intervals per chromosome, thus raising coincidence probability; their raw probabilities must be severely augmented, whereas at the other extreme, very long periods must not. To visualize significant periods, corrected scores (−log [corrected probability]) are plotted along periods (on a logarithmic scale to ease visualization) ranging from 5 kbp to full genome length. Such “periodograms” are shown for all eight species on Fig. 1.

**Figure 2:**
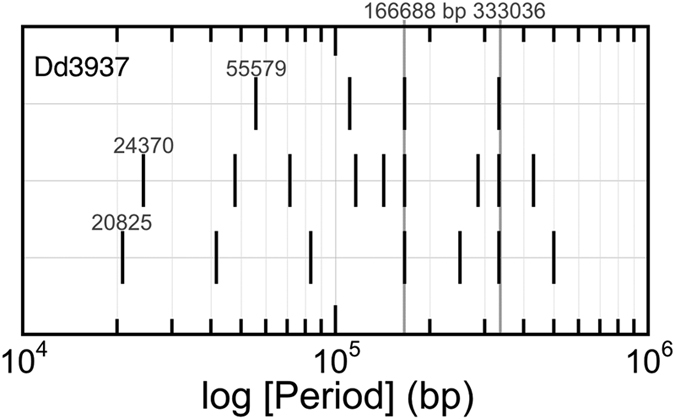
Intra-species harmonic periods. The periodic pattern of the *Dickeya dadantii* 3937 KdgR regulon was analysed^6^,^9^ (Supplementary Table S1). Periods (vertical bars) that were multiples of some lower one (labelled on the leftmost bar) were plotted on the same row in logarithmic scale. Three harmonic families of periods emerged. Furthermore, all families converged on periods 166,688 and 333,036 bp (long grey bars), which themselves are in a ratio of 1.998.

In all species, a few periods show exceptional corrected significance, corresponding to high peaks on their periodogram. Among them, the genomic pattern of the *Dickeya dadantii* 3937 (Dd3937) KdgR regulon displays the richest series of periods (Supplementary Table S1). To assess how distinct these periods really are, they were grouped with their multiples in “harmonic” families. It appears that all these periods fit into three harmonic families. Each family is represented on Fig. 2 as a horizontal thread and labelled after the period of its shortest member. All three families possess two periods in common, 166,688 and 333,036 bp, *i.e.* about 1/30^th^ and 1/15^th^ of the full genome length. Thus, all significant periods in this organism contribute to a single but complex genomic pattern.

**Figure 3:**
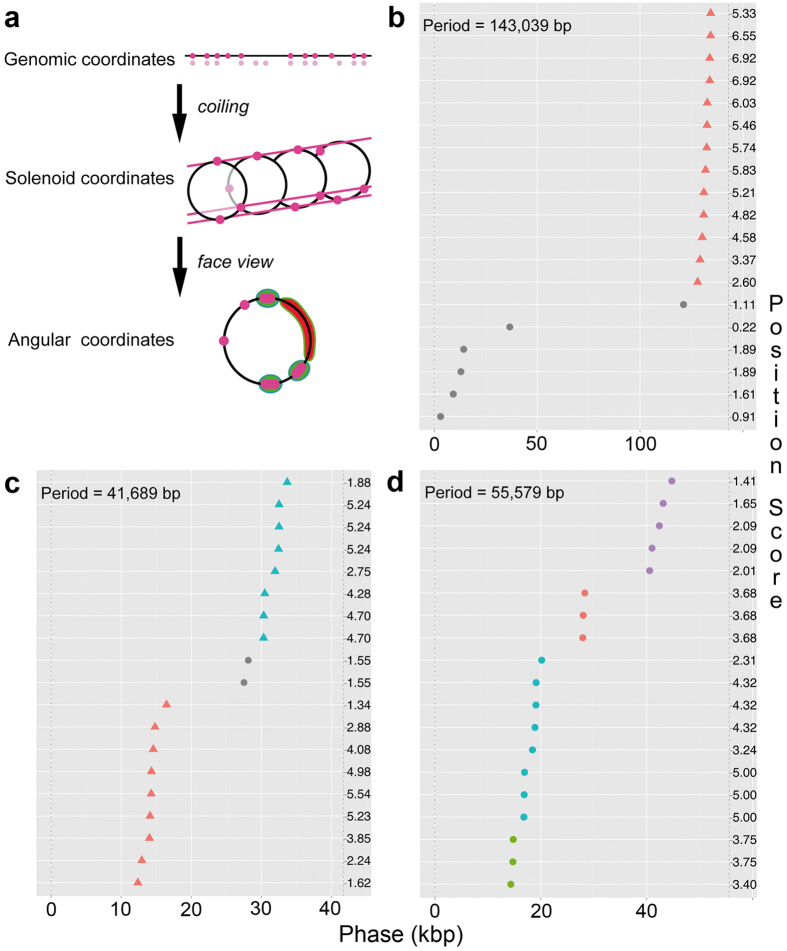
Clustergrams of the top significant periodicities. (**a**) Principle. A set of genomic sites (top, solid dots) imperfectly follows a *P*-periodical pattern (top, blurred dots). These site positions in a solenoidal coordinate of period *P* reveal some alignment properties along the solenoid axis (middle). A projection upon the face view of the solenoid converts the alignments into clustered hot spots (bottom). The scoring function rewards both exceptionally dense (green hot spots) and poor (red) regions (inspired from^9^). (**b–d**) Observations. The periodic pattern of the *D. dadantii* 3937 KdgR regulon was analysed as summarized in (**a**). Because coincident genes would be superimposed in face view, the solenoid was stepwise unfolded along the Y-axis to distinguish all individual genes. This allows to better visualize any alignment as a vertical series. For each of the three harmonic families (Fig. 2), the period *P* with lowest *p*-value is represented here: 143,039 bp (**b**), 41,689 bp (**c**) and 55,579 bp (**d**). The angular coordinates (phases) of all KdgR target genes are plotted on the X-axis between 0 and *P*, delimited by both vertical dashed lines (**b–d**). In-phase gene clusters of minimally three sites were automatically detected by the DBSCAN unsupervised clustering algorithm^6^. They are highlighted with different colours. Isolated genes are plotted as grey dots. Individual gene position scores are shown on the right side. The higher this score, the stronger this gene position contributes to the collective periodicity^9^.

To quantify and visualize individual and overall periodicity, the phases of the KdgR targets with respect to each significant period (Fig. 1) were plotted according to the principle described in Fig. 3a. On Fig. 3b–d, three such “clustergrams” illustrate the three harmonic families of Dd3937 depicted on Fig. 2. Quasi-vertical alignments denote in-phase clustering of genes, *i.e.* their periodical arrangement along the linear genome. For any significant period, the automated procedure detects clusters and highlights them with different colours. It appears that a majority of the regulon targets are periodically aligned in one (Fig. 3b), two (Fig. 3c) or four (Fig. 3d) clusters. To extend the periodicity analysis to all studied species, the clustergrams for their top significant period were computed (Supplementary Fig. S1). Despite the wide range of periods which happen to rank highest in the various species, they all show an alignment of the majority of the KdgR targets in 1–4 clusters. Note that the stringent clustering algorithm applied here sometimes distinguish clusters that have rather close phases.

To map periodic regions at a finer scale, the eight genomes were probed by applying the same periodicity detection algorithm^9^, but now to a sliding window. In subsequent steps, any significantly periodic window is automatically extended until its significance is about to drop. Thus, this self-adaptive sliding window algorithm maximizes the sizes of significantly periodic regions, but does not assume *a priori* that the same period characterizes the full genome^6^. On the example of the Dd3937 KdgR regulon of 15 sites, Supplementary Fig. S2 displays the 11 genome segments that show a strongly periodic pattern involving at least 13 islands out of 15. The upper four segments gathered in panel (a) point to the 143-kbp period—or its double—already detected as the most significant one on the genome taken as a whole (Supplementary Table S1). Each segment covers most of the genome, and their union covers it entirely. Panels (b,c) show segments covering most of the genome with periods already detected as highly significant during the whole-genome analysis (Supplementary Table S1). Panel (b) corresponds to a 499-kbp periodic region. Panel (c) shows the 83-, 167-, 333-kbp pivotal family that was harmonic to all periods (Fig. 2); these three segments together cover all 15 KdgR regulated sites. By contrast, panels (d,e) show periodic regions that were below detection threshold in the whole-genome analysis (Supplementary Table S1). Panel (d) corresponds to a 237-kbp period and the union of both segments covers 14 sites. Panel (e) shows a 417-kbp periodic segment of 13 sites. These seemingly new periods actually belong to the 24,370-bp (d) and 20,825-bp (e) harmonic families of Fig. 2. In sum, for all studied genomes, periodic regions appear to typically extend over the whole genome, even though this sliding window analysis, unlike the previous whole-genome analysis, does not presume the size of the periodic segment.

**Figure 4:**
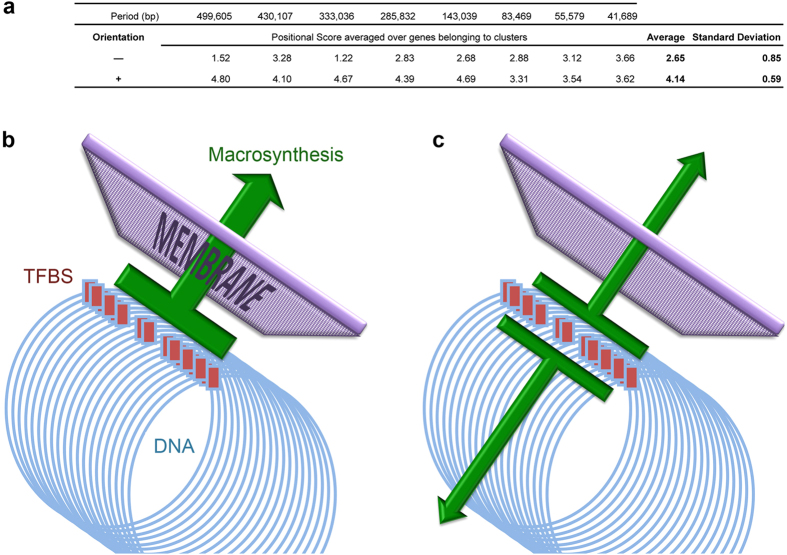
Transcriptional co-orientation of genes in periodic clusters. (**a**) Analysis of all automatically generated clustergrams for the *D. dadantii* 3937 KdgR regulon. For each cluster, the genes transcribed from the (+) or the (−) DNA strand were separately scored by their individual position score as shown *e.g.* on Fig. 3. In the rightmost columns, the average position score of clustered genes is reported separately for either orientation, together with standard deviation. (**b**) Transcriptional co-orientation. A majority of the genes encoding the type II secreted proteins are transcribed from the same DNA strand, thus favoring a coherent flux of neosynthesized pectinases through the membrane. Co-orientation is observed in species Dd3937, DE586, PwWPP163, and possibly Ye8081, PcPC1. (**c**) Lack of co-orientation. Genes are transcribed evenly from both DNA strands. The flux of macromolecular neosynthesis is split in two parts. No co-orientation is found in species Dsp703, DE1591, PaSCRI193.

As one possible interpretation of the observed genome patterns is that they favor intracellular co-localization and assembly of the pathogenic apparatus, further coherence was sought in the transcriptional orientation of genes belonging to a cluster. For each clustergram of the Dd3937 KdgR regulon, corresponding to one period, the individual position scores of all clustered genes were averaged separately for either transcriptional orientation noted (−) and (+) (Fig. 4a). Of eight clustergrams, the average scores are comparable in one case only (period 41,689 bp); in all other seven cases, the (+) averages are higher than the (−) ones. On average, over all Dd3937 clustergrams (two rightmost columns), the (+) category is markedly above the (−) one. These results suggest that transcriptional co-orientation is privileged in periodic clusters. Among the few genes that are not co-oriented with their cluster, most encode sugar transporters or intracellular enzymes of pectin catabolism (KdgK, KdgT, KdgR RhiT, RhiN), which therefore are non-secreted proteins. To quantify and extend this analysis to all eight species, the bias towards transcriptional co-orientation was measured in each cluster by its hypergeometric probability (Supplementary Fig. S3). Co-orientation was observed to be significant for at least one cluster in four species: Dd3937, DE586, PwWPP163, and Ye8081 (the latter after artificially splitting its single cluster in two at its midpoint). PcPC1 displays four non-significant clusters which collectively might constitute a significant bias. Hence, depending on the scoring method, three to five species out of eight show transcriptional co-orientation in the periodic clusters of the pathogenicity genes. However, evidence of co-orientation and absence thereof could not be ascribed to pathogenicity level, geographic origin or phylogenic relation.

## Discussion

In sum, the virulence determinants of type II secretion follow a common principle of genome organization across three genera. Beyond proximity relations that had led to the concept of pathogenicity island, these determinants also show periodicity relations extending a single but complex pattern over the entire genome, thus buttressing the notion of organized pathogenicity archipelago.

Recognition of this new organization level suggests answers to the three issues of co-regulation, co-action and co-transfer. Firstly, periodic genome layouts have been shown to favor DNA folding into solenoidal conformations that spatially cluster co-regulated genes^21^. This spatial clustering had been shown to optimize transcriptional control (*e.g.*, 70-fold for the *lactose* operon^22^,^23^). It is thus probable that the observed genome layout favors efficient co-regulation of the pathogenicity genes^4^,^7^,^24^. Secondly, pathogenic co-action appears to be favored by a novel two-pronged mechanism that has the potential to focus in space and time the abundant secreted enzymes whose joint activities are required for pectin degradation (Fig. 4b,c). Indeed, in bacteria proteins tend to be found close to their encoding gene^25^,^26^, a probable consequence of co-transcriptional translation and of macromolecular crowding. Thus, spatially clustered genes would tend to express grouped proteins. The transcriptional co-orientation observed in half of the species would further focus the macrosynthetic flux towards the membrane translocation pores^27^. It is tempting to speculate that this novel two-pronged mechanism may functionally compensate for the lack, in type II secretion, of a specialized type III needle called “injectisome”^28^ that has sometimes been proven to secrete proteins at localized areas of the bacterium^29^. Thirdly, it may be speculated here that recombination events leading to co-transfer of cognate genes could sometimes occur on the basis of 3-D neighbourhood, in addition to 1-D proximity.

Among the plausible consequences of this new organization level, firstly, it reunifies the genomic description of currently scattered pathogenicity traits, thus facilitating their detection in recently sequenced strains. Secondly, it has been shown that, in addition to the consensus transcription factor binding sequence, gene respective positioning was an effective criterion to predict new co-functional genes by machine learning^30^; in the same vein, the prediction of additional virulence genes should henceforth be facilitated by applying the principle of gene respective positioning. Thirdly, a classical approach to secure DNA production in gene foundries^31^ is to screen customers’ requests against a database of pathogenic DNA sequences^32^; this screening procedure could now be strengthened to encompass a more accurate description of multipartite pathogenic sequences, by including their positional interrelations. Finally, these observations open the gate to new approaches to attenuate pathogenicity traits in bacterial pathogens in case their full inactivation is not desired, *e.g.* for vaccination purposes. For instance, if the virulence genes’ promoter structure/function relation is not mastered in a little-described or emerging pathogen, virulence factors production could still be modulated by lessening their periodicity/proximity relations and transcriptional co-orientations under model guidance.

## Methods

### Strains and genomes

All bacteria used in this study have a unique circular chromosome and no accessory plasmid, except *Yersinia enterocolitica* subsp. *enterocolitica* 8081 which harbors the 67.7-kb plasmid pYVe8081. Plasmid pYVe8081 (GenBank accession number AM286416.1) was excluded from our analysis. The accession numbers of the complete genomes used in this study are indicated in Table 1. Several sequenced *Dickeya* strains have been reclassified and public databases fail to be updated accordingly: Ech586 was moved from *D. dadantii* to *D. zeae*, Ech703 from *D. dadantii* to *D. paradisiaca* and Ech1591 from *D. zeae* to *D. chrysanthemi*^33^.

### Datasets

The genes regulated by KdgR (KdgR regulon) were determined by constructing a position weight matrix for the KdgR binding site with known *D. dadantii* targets using the motif discovery MEME tool^34^. The sequence logo for the KdgR binding site is shown as Supplementary Fig. S4. This 17-bp long motif was supplied to the motif alignment MAST tool^35^ for scanning the selected genomes. A position was classified as a positive hit when the p-value was less than 0.0001. Most targets detected using this cut-off were identified as being involved in the pectin catabolic pathway. This result demonstrates the specificity of using the KdgR motif for prediction. For all organisms, this procedure was used to obtain the initial dataset that consisted of a list of genes and their genomic positions. In all cases, a genomic position is defined by the gene’s translation start site, which is very close to the KdgR transcription factor binding site but more amenable to systematic analysis.

### Pattern analysis

If in a dataset, *n* sites had very close genomic positions, they would contribute *n* times quasi-equivalent distances to any distant genomic site of the same dataset. Such repetitious distances would unduly bias the analysis. To avoid this neighbourhood bias, an initial dataset is reduced in two steps. First, each operon is reduced to its upstream cistron which is closest to the promoter. Second, each set of neighbour (within 2 kbp) genes/operons is replaced by a single site located at their mean position (*-prox* in Table 1). The genomes are scanned for periods ranging from 5,000 bp to full genome length. For each scanned period, periodicity of the positions of sites is assessed. To this end, randomized sets of positions (same number, identical chromosome length) are generated and their periodicity measured, until 10 random sets give equal or better periodicity level than the observed dataset. For instance, if it took 10^4^ randomized sets to observe 10 equal or better periodicity levels, the probability attributed to this observed periodicity is 10/10^4^ = 10^−3^. This “raw” probability is then corrected for multiple testing: the shorter the period, the larger the number of intervals per genome, hence the larger the chances to observe coincident positions. For “periodograms” such as on Fig. 1, the cologarithm of this corrected probability is plotted on the Y-axis (*e.g.*, the label “3” corresponds to a probability of 10^−3^). High peaks denote periodicity. Where the peak(s) is on the higher end of the spectrum, *i.e.* for periods close or equal to full genome length or a large fraction of it (one half, one third etc.), it denotes a proximal arrangement of the genes around the circular chromosome. Such a proximity is still far beyond the immediate neighbourhood cases which have been removed prior to analysis (*-prox* in Table 1). Indeed, one advantage of the algorithm used here is that it allows to detect both proximity and periodicity in a single pass^9^.

### Cluster analysis

For any period giving a significant pattern, a “clustergram” may be generated. A clustergram visualises as vertical alignments clusters of genomic sites after plotting their modulo-period coordinate (phase) on the X-axis and their phase ranking on the Y-axis (Fig. 3a). This clustering is quantified by applying an unsupervised clustering algorithm known in data sciences as DBSCAN^6^. DBSCAN requires two parameters, the minimum size of the cluster, and the minimum distance between phases which is set as the ratio of the average intergenic size to the period; this ratio is normalised by a single parameter called the clustering exponent.

### Availability

The software suite implementing this algorithm, “*GREAT:SCAN:patterns*” (“GREAT” stands for “Genome REgulatory and Architecture Tools”), is available online^36^ from abSYNTH, the iSSB technological platform of synthetic biology. The corresponding protocol for genome analysis is described in detail elsewhere^6^. The parameters were set as follows, *e.g.* for Dd3937: GREAT:SCAN:patterns # avgGene: 1000, clustExp: 0, clustSize: 3, infile: Dd3937_pos, length: 4922776, mapSelect: 0.001, perRange: 5000, 4922776, pvSelect: 0.05, pvThres: 0.01, setCoords: 1e + 06, 2e + 06, 3e + 06, 4e + 06, setTicks: 1, 2, 3, 4, textSize: 12, title: Dd3937, uniqID: Dd3937_scm.

## Additional Information

**How to cite this article**: Bouyioukos, C. *et al.* From multiple pathogenicity islands to a unique organized pathogenicity archipelago. *Sci. Rep.* **6**, 27978; doi: 10.1038/srep27978 (2016).

## Supplementary Material

Supplementary Information

## Figures and Tables

**Table 1:** Bacterial pathogens under scrutiny.

Short name	Chromosome length (bp)	Regulon size		Full name	GenBank accession no. complete chromosome
		full	-prox		
Plant pathogens					
Dd3937	4,922,776	19	15	*Dickeya dadantii* 3937	CP002038.1
DE586	4,818,394	15	10	*Dickeya zeae* Ech586	CP001836.1
Dsp703	4,679,450	13	9	*Dickeya paradisiaca* sp703	CP001654.1
DE1591	4,813,854	18	12	*Dickeya chrysanthemi* Ech1591	CP001655.1
PaSCRI193	5,064,019	20	13	*Pectobacterium atrosepticum* SCRI193	BX950851.1
PcPC1	4,862,913	20	15	*Pectobacterium carotovorum* PC1	CP001657.1
PwWPP163	5,063,892	20	16	*Pectobacterium wasabiae* WPP163	CP001790.1
Human pathogen					
Ye8081	4,615,899	11	9	*Yersinia enterocolitica* 8081	AM286415.1

Short (column 1) and long (column 5) names of the eight genera/species/strains used in this study are shown, as well as their chromosome length (column 2) and GenBank accession number (column 6). Columns 3 and 4 show their number of known target genes in the KdgR regulon, before (full) or after (-prox) replacing neighbour genes by a single site at the mean position of the original loci. Neighbour genes are defined as closer than two times the average gene-to-gene distance of the studied genome, taken here to be 1,000 bp. Further analysis is systematically carried out on the -prox dataset (the set of pathogenicity islands) to avoid artificially boosting statistical significance.

## References

[b1] Huynen M. & Bork P. Measuring genome evolution. Proc Natl Acad Sci USA 95, 5849–5856 (1998). 9600883 10.1073/pnas.95.11.5849 PMC34486

[b2] Tamames J. Evolution of gene order conservation in prokaryotes. Genome biology 2, 1178 (2001). 10.1186/gb-2001-2-6-research0020 PMC33396 11423009

[b3] Rocha E. P. C. The organization of the bacterial genome. Annu Rev Genet 42, 211–33 (2008). 18605898 10.1146/annurev.genet.42.110807.091653

[b4] Képès F. Periodic transcriptional organization of the *E. coli* genome. Journal of molecular biology 340, 957–964 (2004). 15236959 10.1016/j.jmb.2004.05.039

[b5] Képès F. Periodic epi-organization of the yeast genome revealed by the distribution of promoter sites. Journal of Molecular Biology 329, 859–865 (2003). 12798677 10.1016/s0022-2836(03)00535-7

[b6] Bouyioukos C., Elati M. & Képès F. Protocols for probing genome architecture of regulatory networks in hydrocarbon and lipid microorganisms. In Hydrocarbon and Lipid Microbiology Protocols (eds McGenity, Timmis & Nogales) (Springer-Verlag Berlin Heidelberg, 2015).

[b7] Junier I., Hérisson J. & Képès F. Genomic organization of evolutionarily correlated genes in bacteria: limits and strategies. Journal of molecular biology 419, 369–386 (2012). 22446685 10.1016/j.jmb.2012.03.009

[b8] Wright M. A., Kharchenko P., Church G. M. & Segrè D. Chromosomal periodicity of evolutionarily conserved gene pairs. Proc Natl Acad Sci USA 104, 10559–10564 (2007). 17563360 10.1073/pnas.0610776104 PMC1890563

[b9] Junier I., Hérisson J. & Képès F. Periodic pattern detection in sparse boolean sequences. Algorithms for molecular biology: AMB 5, 31 (2010). 20831781 10.1186/1748-7188-5-31 PMC2949599

[b10] Gal-Mor O. & Finlay B. B. Pathogenicity islands: a molecular toolbox for bacterial virulence.Cellular microbiology 8, 1707–1719 (2006). 10.1111/j.1462-5822.2006.00794.x 16939533

[b11] Wilson M., McNab R. & Henderson B. Bacterial Disease Mechanisms: An Introduction to Cellular Microbiology 95 (Cambridge University Press, 2002).

[b12] Peters J., Wilson D. P., Myers G., Timms P. & Bavoil P. M. Type III secretion a la *Chlamydia*. Trends in microbiology 15, 241–251 (2007). 17482820 10.1016/j.tim.2007.04.005

[b13] Reverchon S. & Nasser W. *Dickeya* ecology, environment sensing and regulation of virulence programme. Environmental microbiology reports 5, 622–636 (2013). 24115612 10.1111/1758-2229.12073

[b14] Charkowski A. *et al.* The role of secretion systems and small molecules in soft-rot Enterobacteriaceae pathogenicity. Annual review of phytopathology 50, 425–449 (2012). 10.1146/annurev-phyto-081211-173013 22702350

[b15] von Tils D., Bladel I., Schmidt M. A. & Heusipp G. Type II secretion in *Yersinia*-a secretion system for pathogenicity and environmental fitness. Frontiers in cellular and infection microbiology 2, 160 (2012). 23248779 10.3389/fcimb.2012.00160 PMC3521999

[b16] Korotkov K. V., Sandkvist M. & Hol W. G. The type II secretion system: biogenesis, molecular architecture and mechanism. Nature reviews Microbiology 10, 336–351 (2012). 22466878 10.1038/nrmicro2762 PMC3705712

[b17] Robert-Baudouy J. *et al.* Pectic enzymes of *Erwinia chrysanthemi* regulation and role in pathogenesis. In Plant-Microbe Interactions Vol. 5 (eds Stacey G. & Keen N. T.) 221–268 (The American Phytopathological Society, St Paul, Minnesota, 2000).

[b18] Reverchon S., Nasser W. & Robert-Baudouy J. Characterization of *kdgR*, a gene of *Erwinia chrysanthemi* that regulates pectin degradation. Molecular microbiology 5, 2203–2216 (1991). 1840643 10.1111/j.1365-2958.1991.tb02150.x

[b19] Nasser W., Reverchon S., Condemine G. & Robert-Baudouy J. Specific interactions of *Erwinia chrysanthemi* KdgR repressor with different operators of genes involved in pectinolysis. Journal of molecular biology 236, 427–440 (1994). 8107132 10.1006/jmbi.1994.1155

[b20] Rodionov D. A., Gelfand M. S. & Hugouvieux-Cotte-Pattat N. Comparative genomics of the KdgR regulon in *Erwinia chrysanthemi* 3937 and other gamma-proteobacteria. Microbiology 150, 3571–3590 (2004). 15528647 10.1099/mic.0.27041-0

[b21] Junier I., Martin O. & Képès F. Spatial and topological organization of DNA chains induced by gene co-localization. PLoS computational biology 6, e1000678 (2010). 20169181 10.1371/journal.pcbi.1000678 PMC2820526

[b22] Dröge P. & Müller-Hill B. High local protein concentrations at promoters: strategies in prokaryotic and eukaryotic cells. Bioessays 23, 179–183 (2001). 11169591 10.1002/1521-1878(200102)23:2<179::AID-BIES1025>3.0.CO;2-6

[b23] Vilar J. M. & Leibler S. DNA looping and physical constraints on transcription regulation. Journal of molecular biology 331, 981–989 (2003). 12927535 10.1016/s0022-2836(03)00764-2

[b24] Képès F. & Vaillant C. Transcription-based solenoidal model of chromosomes. ComPlexUs 1, 171–180 (2003).

[b25] Miller O. L. Jr., Hamkalo B. A. & Thomas C. A. Jr. Visualization of bacterial genes in action. Science 169, 392–395 (1970). 4915822 10.1126/science.169.3943.392

[b26] Montero Llopis P. *et al.* Spatial organization of the flow of genetic information in bacteria. Nature 466, 77–81 (2010). 20562858 10.1038/nature09152 PMC2896451

[b27] Matsumoto K., Hara H., Fishov I., Mileykovskaya E. & Norris V. The membrane: transertion as an organizing principle in membrane heterogeneity. Frontiers in microbiology 6, 572 (2015). 26124753 10.3389/fmicb.2015.00572 PMC4464175

[b28] Costa T. R. *et al.* Secretion systems in Gram-negative bacteria: structural and mechanistic insights. Nature reviews Microbiology 13, 343–359 (2015). 25978706 10.1038/nrmicro3456

[b29] Jaumouille V., Francetic O., Sansonetti P. J. & Tran Van Nhieu G. Cytoplasmic targeting of IpaC to the bacterial pole directs polar type III secretion in Shigella. The EMBO journal 27, 447–457 (2008). 18188151 10.1038/sj.emboj.7601976 PMC2234337

[b30] Elati M. *et al.* PreCisIon: PREdiction of CIS-regulatory elements improved by gene’s positION. Nucleic acids research 41, 1406–1415 (2013). 23241390 10.1093/nar/gks1286 PMC3561985

[b31] Kelle A. Security Issues Related to Synthetic Biology. In Synthetic Biology (eds Schmidt, Kelle, Ganguli-Mitra, Vriend) (Springer Science+Business Media B.V., 2009).

[b32] Bugl H. *et al.* DNA synthesis and biological security. Nature biotechnology 25, 627–629 (2007). 10.1038/nbt0607-627 17557094

[b33] Marrero G., Schneider K. L., Jenkins D. M. & Alvarez A. M. Phylogeny and classification of *Dickeya* based on multilocus sequence analysis. Int J Syst Evol Microbiol 63, 3524–39 (2013). 24003072 10.1099/ijs.0.046490-0

[b34] Bailey T. L. & Elkan C. Fitting a mixture model by expectation maximization to discover motifs in biopolymers. Proceedings of the Second International Conference on Intelligent Systems for Molecular Biology. 28–36 (AAAI Press, Menlo Park, California, 1994). 7584402

[b35] Bailey T. L. & Gribskov M. Combining evidence using p-values: application to sequence homology searches. Bioinformatics 14, 48–54 (1998). 9520501 10.1093/bioinformatics/14.1.48

[b36] Bouyioukos C., Bucchini F., Elati M. & Képès F. GREAT: a web portal for Genome REgulatory Architecture Tools. Nucleic Acids Research (Web Server Issue). (Advance Access published May 5, 2016). 10.1093/nar/gkw384 PMC4987929 27151196

