# Supplementary material for: From multiple pathogenicity islands to a unique organized pathogenicity archipelago
Source: Sci Rep. 2016 Jun 15;6:27978. doi: 10.1038/srep27978 (PMC4908373; doi:10.1038/srep27978)
Supplement: Supplementary Information [file srep27978-s1.pdf]

From multiple pathogenicity islands to a unique organized pathogenicity archipelago

Costas Bouyioukos[1], Sylvie Reverchon[2] & François Képès[1,3]

[1] institute of Systems and Synthetic Biology, Genopole, CNRS, Univ. Evry, 91000 Evry, France.

[2] Univ Lyon, Université Lyon 1, INSA-Lyon, CNRS UMR5240, MAP, F-69622 VILLEURBANNE, France.

[3] Department of BioEngineering, Imperial College London, United Kingdom.

SUPPLEMENTARY INFORMATION

Supplementary Table S1 | Most significant periods observed for the *D. dadantii* 3937 KdgR regulon

Supplementary Figure S1 | Clustergrams of the KdgR regulon for all eight genomes

Supplementary Figure S2 | Genomic map of periodic regions

Supplementary Figure S3 | Transcriptional orientation of genes in periodic clusters of all species

Supplementary Figure S4 | Sequence logo for the KdgR-binding motif

Supplementary Table S1 | Top significant periods observed for the *D. dadantii* 3937 KdgR regulon

Period (bp)	Corrected Probability	Raw	Period (bp)	Corrected Probability	Raw
20825	1.7E-02	7.3E-05	**333036**	1.0E-02	2.9E-03
24370	3.4E-02	1.7E-04	**430107**	1.0E-02	5.5E-04
**41689**	8.9E-04	7.5E-06	**499605**	2.1E-03	2.5E-04
47730	4.8E-02	4.6E-04	633930	1.2E-02	3.9E-04
**55579**	1.0E-02	1.3E-04	816612	1.8E-02	3.1E-03
71461	1.9E-02	2.8E-04	931481	3.5E-02	2.7E-03
**83469**	5.5E-03	9.4E-05	984555	4.1E-02	8.3E-03
111193	3.8E-02	8.6E-04	1146096	1.8E-02	5.7E-03
116291	4.5E-02	1.1E-03	1216038	2.8E-02	7.1E-03
**143039**	4.3E-06	1.3E-07	1278411	2.3E-03	6.0E-03
166688	3.1E-02	1.0E-03	1633900	2.3E-02	7.7E-03
250340	3.8E-02	1.1E-03	2422146	1.2E-02	5.9E-03
**285832**	6.7E-05	3.9E-06	4625701	1.3E-02	7.7E-03

Significant periods were automatically extracted by the "*GREAT:SCAN*" suite from the periodogram of Fig. 1 (upper-left panel, species "Dd3937"), where they appear as peaks above the threshold set at probability 5.10^{-2}. These significant periods are ranked by size in the left column. The probability that a similar or superior periodicity level could be achieved with a randomized set of positions is shown in the right column. It is corrected for period-dependance in the middle column, which therefore displays the final measure of period significance. Periods highlighted in bold have a corrected probability under 10^{-2} (tantamount to a corrected significance score above 2) and are studied further. Periods shown in grey are above 10% of full genome length and are thus partly or totally reflecting proximity patterns; as only periodic patterns are of interest, they are not further considered.

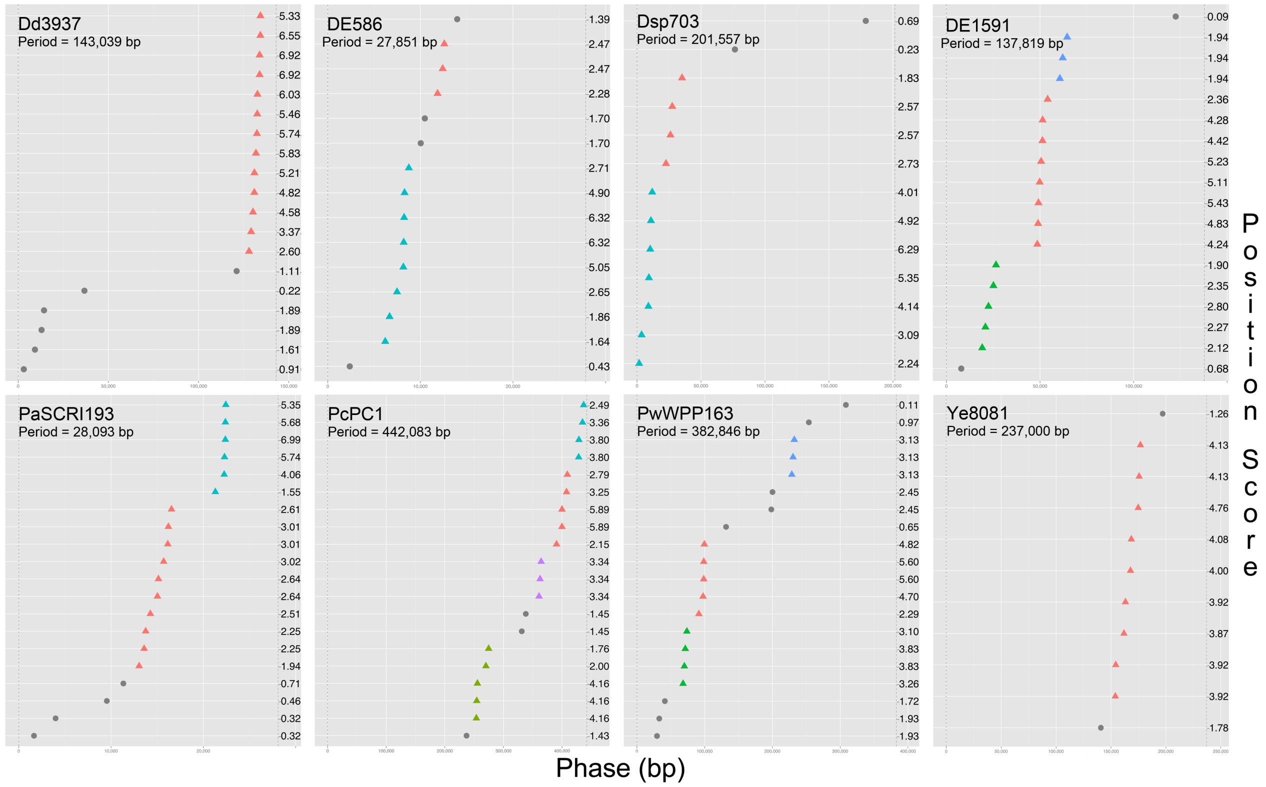

Supplementary Figure S1 | Clustergrams of the KdgR regulon for all eight genomes. Periodicity analysis was conducted with the GREAT:SCAN suite as before. The clustergram for the top significant period of each species (as labelled in the upper left corner) is represented here. A gene from the dataset corresponds to one dot. Genes are positioned with respect to the X-axis according to their phase position with respect to the period considered. Thus, any vertical quasi-alignment denotes a group of periodically-disposed genes and is labelled with a distinct colour. Individual gene position scores are provided on the right side. A high score indicates a gene that contributes strongly to the collective periodicity. Legend as in Fig. 3.

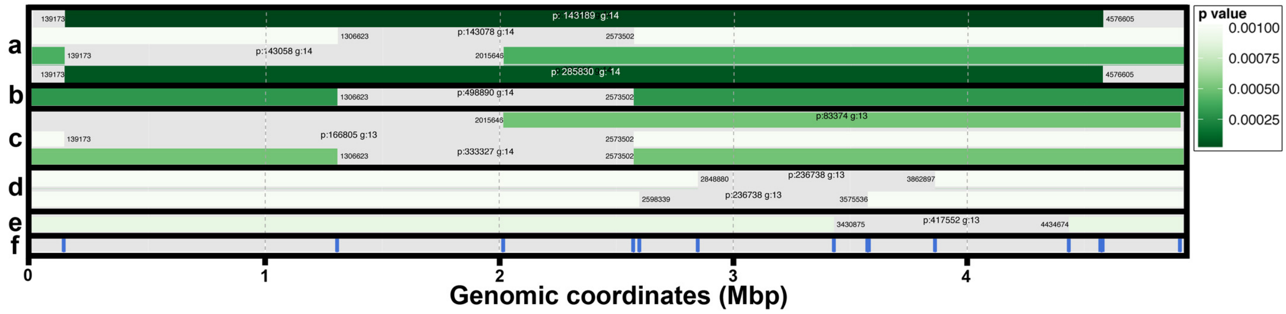

Supplementary Figure S2 | Genomic map of periodic regions. The periodic pattern of the *D. dadantii* 3937 KdgR regulon was analysed as before, except that the analysis was applied to a sliding window running along the genome, rather than to the genome taken as a whole. By maximally extending the periodic windows thus detected, contiguous regions with a significant corrected score of periodicity are mapped. **f,** positions of the 15 non-neighbor sites (blue bars); some are too close to be distinguished at this scale. **a-e**, the 11 segments of the genome that contain at least 13 periodic sites out of 15 are depicted, grouped in five panels according to period categories (see main text). The colour code corresponds to the corrected probability for this period (legend in right panel). For each significant segment appear (small font) its end coordinates, period value in bp (p:) and number of sites/genes (g:). The X-axis displays the coordinates for the full genome length.

Species	Dd3937		DE586		Dsp703		DE1591		PaSCRI193		PcPC1		PwWPP163		Ye8081	
Period (bp)	333,036		312,003		201,557		311,658		298,859		442,083		316,493		237,000	
Cluster	Size	P-value	Size	P-value	Size	P-value	Size	P-value	Size	P-value	Size	P-value	Size	P-value	Size	P-value
	9	7.7 E-3	5	4.4 E-1	7	2.0 E-1	8	3.2 E-1	6	3.9 E-1	5	3.8 E-1	6	3.6 E-1	9	5.5 E-1
	3	3.0 E-1	4	3.2 E-2	4	4.1 E-1	3	2.7 E-1	5	3.2 E-1	5	3.8 E-1	3	7.4 E-2		
			3	3.6 E-1							4	1.9 E-1	3	1.4 E-1		
											3	1.4 E-1				
	12 in 19	2.3 E-3	12 in 15	5.1 E-3	11 in 13	8.0 E-2	11 in 18	8.5 E-2	11 in 20	1.3 E-1	17 in 20	4.1 E-3	12 in 20	3.8 E-3	9 in 11	5.5 E-1
															5	6.5 E-2
															4	4.5 E-2
															9 in 11	3.0 E-3

Supplementary Figure S3 | Transcriptional orientation of genes in periodic clusters of all species. In each of the eight species (top row), the KdgR regulon clustergram for the pivot period (second row) determined as in Fig. 2 was analysed cluster by cluster. For each cluster in the species clustergram, the genes transcribed from the (+) or the (-) strand were scored. The bias towards transcriptional co-orientation was measured by its hypergeometric probability. For each species, the number of genes per cluster is shown in the left column, and the hypergeometric probability in the right column. Bold, probabilities under 10^{-1}. In the bottom row, the number of genes from all clusters and the total number of genes in the regulon are provided in the left column. The product of all above probabilities is in the right column. If the clusters of one clustergram were independent, this product would equal the probability of co-occurrence of co-oriented clusters for the given period. However, as most genes belong to one cluster or another, independence cannot be assumed; therefore, this product does not equal this co-occurrence probability but puts a lower bound on it. Green (red) background, product probability under (over) 10^{-2}. A special case is PcCP1 which has four clusters, each non-significant, but collectively significant. A different case is Ye8081: its single cluster successively contains a stretch of (+) followed by one of (-) sites; splitting this single cluster in the middle yields two significantly co-oriented clusters (bottom three rows).

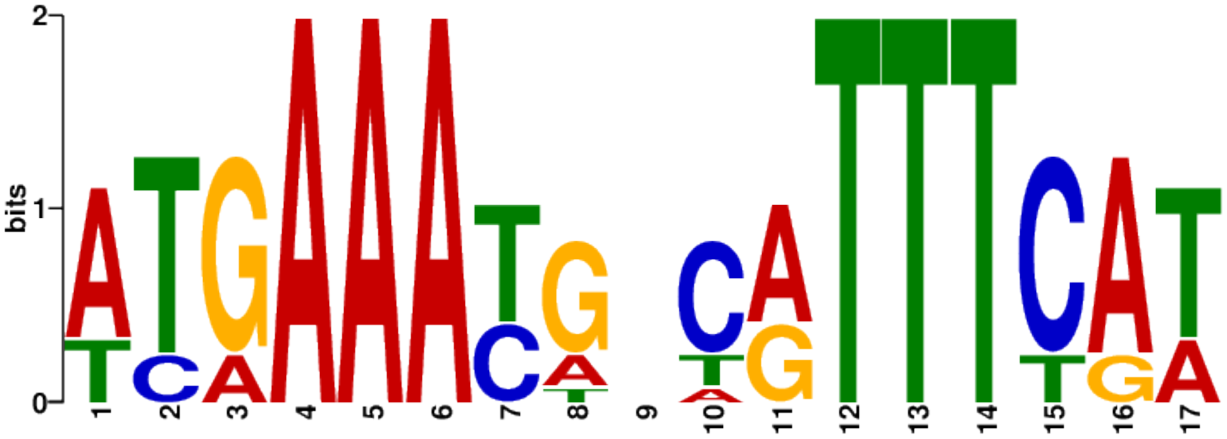

Supplementary Figure S4 | Sequence logo for the KdgR-binding motif. This motif site was drawn using the motif discovery MEME tool[34] by using a training set of known *D. dadantii* KdgR-binding sites.
